# An Avatar-Led Web-Based and SMS Text Message Smoking Cessation Program for Socioeconomically Disadvantaged Veterans: Pilot Randomized Controlled Trial

**DOI:** 10.2196/44503

**Published:** 2023-04-14

**Authors:** Jaimee L Heffner, Megan M Kelly, Erin D Reilly, Scott G Reece, Tracy Claudio, Edit Serfozo, Kelsey Baker, Noreen L Watson, Maria Karekla

**Affiliations:** 1 Fred Hutchinson Cancer Center Seattle, WA United States; 2 VA Bedford Healthcare System Bedford, MA United States; 3 University of Massachusetts Chan Medical School Worcester, MA United States; 4 University of Cyprus Nicosia Cyprus

**Keywords:** embodied agent, tobacco cessation, nicotine dependence, mobile health, mHealth

## Abstract

**Background:**

Despite the declining prevalence of cigarette smoking in the United States, socioeconomically disadvantaged veterans receiving care from the Veterans Health Administration have a high prevalence of smoking. Currently, available treatment options for these veterans focus on tobacco users who are ready to quit and have limited reach. Consequently, there is a great need for accessible, effective smoking cessation interventions for veterans at all levels of readiness to quit smoking.

**Objective:**

To address these needs, we developed Vet Flexiquit, a web-based Acceptance and Commitment Therapy program for veterans, and evaluated its acceptability (primary aim), efficacy, and impact on theory-based change processes relative to the National Cancer Institute’s SmokefreeVET program in a pilot randomized controlled trial.

**Methods:**

Participants (N=49) were randomized 1:1 to receive either the Vet Flexiquit (n=25) or SmokefreeVET (n=24) web program. Both groups received SMS text messages as part of the intervention for 6 weeks. Both interventions are fully automated and self-guided. Primary outcome data were collected at 3 months after the randomization. Self-reported smoking abstinence was biochemically verified using saliva cotinine. Multivariable logistic regression, negative binomial regression, and linear regression models were used to evaluate the association between the treatment arm and outcomes of interest.

**Results:**

Acceptability, as measured by overall treatment satisfaction, was high and similar across treatment arms: 100% (17/17) for Vet Flexiquit and 95% (18/19) for SmokefreeVET. Acceptability, as measured by utilization, was more modest (log-ins: *M*=3.7 for Vet Flexiquit and *M*=3.2 for SmokefreeVET). There were no statistically significant differences between treatment arms for any acceptability measures. Similarly, there were no statistically significant differences between treatment arms in the secondary outcomes of smoking cessation or change in Acceptance and Commitment Therapy’s theory-based processes. In open-ended survey responses, some veterans in both treatment arms expressed interest in having support from a professional or peer to enhance their experience, as well as an expanded SMS text messaging program.

**Conclusions:**

Both programs had high ratings of acceptability, limited utilization, and a similar impact on cessation and cessation processes. Taken together with the qualitative data suggesting that additional support may enhance participants’ experience of both programs, these preliminary findings suggest that the programs may have similar outcomes among veterans who are looking for a digital cessation treatment option and that integrating provider or peer support and enhancing the SMS text messaging program holds promise as a means of boosting engagement and outcomes for both programs.

**Trial Registration:**

ClinicalTrials.gov NCT04502524; https://clinicaltrials.gov/ct2/show/NCT04502524

## Introduction

Cigarette smoking is responsible for over 440,000 deaths annually and causes 32% of all cancer deaths [1,2]. In addition, smoking adds US $193 billion in health care expenditures and productivity losses each year in the United States [3]. The US Veterans Health Administration (VHA), which has ≥1 million enrollees who use tobacco among 8.7 million total enrollees [4], spends US $2.7 billion per year treating smoking-related health problems [5]. Although smoking rates have declined in the United States over the past 50 years, there has been an upturn in smoking among military personnel in the last 2 decades [6], and the prevalence of current smoking among veterans is higher than that among nonveterans [6,7]. The elevated prevalence of smoking is most substantial among male veterans, who comprise the vast majority (94%) of veterans served by the VHA [4]: smoking prevalence among veterans versus nonveterans is 50% versus 35% for ages 18 to 25 years, 46% versus 36% for ages 26 to 34 years, and 32% versus 26% for ages 35 to 49 years [7].

Veterans represent an important subgroup of socioeconomically disadvantaged smokers. The VHA serves an estimated 75% of all low-income and disabled veterans [6]. Although the VHA has always served as a safety-net health care provider in the United States, the Affordable Care Act and health care reform have increased the proportion of VHA users with low socioeconomic status (SES) [8-10]. Only 40% of veterans served by the VHA are in the labor force, and the median household income is only US $35,999 [4]. Consistent with larger US population trends, within the VHA, current smoking is associated with the lowest levels of income, educational attainment, and employment [11].

Smoking cessation treatment approaches currently available to socioeconomically disadvantaged veterans receiving care through the VHA include local options for group and individual counseling as well as centrally managed population health programs such as the VA Quitline, SmokefreeVET text messaging program, Stay Quit Coach app, and SmokefreeVET website. There are several limitations to these program offerings, the most substantial of which are as follows: (1) mismatch in readiness to quit, with existing programs relying on standard treatment approaches that are designed to meet the needs of smokers who are planning to quit in the near future (eg, the next 30 days), which is only approximately 20% of current smokers [12], and (2) low accessibility and engagement, as traditional cessation programs such as group or individual counseling are underresourced to assist even those veterans who are highly motivated to quit [6,13] and have limited reach, with only 0.9% of veteran smokers served by the VHA receiving the US Public Health Service recommendation [14] for intensive (≥10 minutes) cessation support [13]. Digital cessation interventions are more accessible, particularly in low-resource health care systems such as the VHA, but maintaining engagement with digital interventions is a challenge [15] that requires innovative design solutions, as does the problem of low quit rates for existing digital interventions for veterans served through the VHA. For example, in a study of the real-world effectiveness of the SmokefreeVET text messaging program, Christofferson et al [16] found that self-reported 30-day abstinence rates were 4.5% at 3 months after enrollment and 3.7% at 6 months. These limitations of current treatment options are problematic not just for veterans but also for socioeconomically disadvantaged smokers more broadly. These tobacco users are less likely to be planning to quit in the near future, less likely to make a quit attempt, and, when they attempt to quit, less likely to engage with evidence-based treatments that substantially improve cessation outcomes and instead make unaided quit attempts relative to smokers who are not socioeconomically disadvantaged [17,18].

In addition to offering more accessible treatment modalities, newer models of treatment may be better able to address the cessation challenges experienced by socioeconomically disadvantaged veterans. Acceptance and Commitment Therapy (ACT) [19,20] is similar to standard smoking cessation treatment in that it promotes awareness of the cues that trigger smoking behavior but is different in that ACT teaches skills to promote *acceptance* of triggers (eg, mindfulness) rather than trigger *avoidance,* which is not always possible. Empirical support for ACT comes from 11 trials that enrolled over 6000 tobacco users [21-32]. Collectively, these studies support the feasibility and efficacy of ACT relative to pharmacotherapy-only treatments and traditional behavioral treatments, with the majority demonstrating quit rates for ACT that were superior to control group quit rates at short-term (ie, 3 months) and long-term (6-12 months) follow-up. Importantly, ACT is acceptable and shows evidence of efficacy for smokers with mental health conditions including mood disorders and posttraumatic stress disorder [33-36], which are highly prevalent among veterans [13] and among smokers with low SES [37], may interfere with quitting [38], and are not addressed in standard cessation treatments. Taken together, these findings suggest that ACT is at least as effective, if not more effective, than standard treatment approaches and that it uniquely addresses some of the challenges to smoking cessation among populations with low SES.

All but one [32] of the previous trials of ACT for smoking cessation restricted enrollment to people who were ready to quit smoking, and the intervention focused on action-oriented strategies for coping with cravings. However, ACT can also be used to motivate people at lower levels of quit readiness by placing greater emphasis on the ACT components of awareness and enactment of personal values at the outset of the treatment.

Karekla et al [32] developed the first web-based ACT program designed for smokers at all stages of readiness to quit. The foundational avatar-led ACT program, Flexiquit, was innovative in its use of gamification, persuasive technology, and avatar coaches to motivate user interaction [39]. Flexiquit was evaluated in 105 university student smokers in Cyprus, aged 18 to 28 years (mean 22.50, SD 2.56 years; 45 females). Participants were randomly assigned in a 2:1 ratio to either the Flexiquit group (n=70) or a waitlist control group (n=35). Results indicated that the program content was highly acceptable, even among those who had no intention to quit [40]. Overall, 89% said that the content was easy to understand, 74% found it very interesting, and 60% completed all 6 sessions. Compared with the waitlist control group, quit rates for Flexiquit were significantly higher at the end of treatment (29% for Flexiquit vs 11% for waitlist control), demonstrating a strong signal for efficacy [32].

In a subsequent study [41], we tailored the Flexiquit program for sexual and gender minority young adults in the United States and evaluated it in a single-arm pilot trial (n=22). The new program—called EQQUAL (Empowered, Queer, Quitting, and Living)—had high acceptability; 93% of the participants were satisfied overall, 100% reported that the program was easy to navigate, and 100% said that they felt clearer about how they might quit because of using the EQQUAL program. On the acceptability outcome of program use, the average number of log-ins was 5.5 (SD 3.6), the average number of sessions completed was 3.1 (SD 2.6), and 39% completed all 6 sessions. The biochemically confirmed 7-day point prevalence abstinence (PPA) at the end of treatment was 22.7%, which is 3 times higher than the only other targeted digital smoking cessation intervention for sexual and gender minority young adults [42].

We followed a user-centered design process to develop a veteran-tailored version of the Flexiquit program, called Vet Flexiquit, to enhance current smoking cessation treatment offerings within the VHA. Vet Flexiquit’s use of gamification and avatar coaches could increase engagement, and ultimately cessation outcomes, among veterans at all levels of quit readiness by making the experience pleasurable through novelty and interactivity. In addition, an avatar coach, represented as a supportive peer who was able to quit successfully, can instill hope by sharing personal quitting narratives. In addition, the avatar coach, as the embodiment of a supportive peer, fits the VHA’s organizational values. The VHA is the country’s largest employer of peer support specialists, recognizing the unique importance of peer support to veterans—particularly those with mental health conditions—who may feel isolated and unable to relate as well to nonveterans.

The primary aim of this study was to compare the relative acceptability of Vet Flexiquit versus SmokefreeVET among socioeconomically disadvantaged US veterans, as indicated by treatment satisfaction and objective measures of website utilization. The secondary aim was to preliminarily evaluate the effects of Vet Flexiquit versus SmokefreeVET on quit attempts and abstinence rates as well as readiness to quit and acceptance of smoking triggers—ACT’s theory-based mechanism of change**.**

## Methods

### Participants

Participants were recruited from the VA Bedford Healthcare System as well as via flyers and outreach to VA providers in the Boston metro area. Inclusion criteria were as follows: (1) US veteran; (2) age ≥18 years; (3) meets the VHA national threshold for no-cost health care based on income or disability status; (4) current smoker, averaging at least 5 cigarettes per day for the last 30 days; (5) weekly internet access for the next 3 months; (6) current use of a personal email address to receive the link to their assigned website; and (7) willingness to complete all study activities and to receive study-related SMS text messages. Exclusion criteria were as follows: (1) currently taking part in any other smoking cessation treatment including pharmacotherapies, counseling, or other digital cessation programs; (2) recent (past 30 days) substance use disorder, suicidal ideation, or psychiatric hospitalization; (3) previous participation in the treatment development stage of Vet Flexiquit; (4) prior use of the SmokefreeVET website; (5) member of the same household as another research participant; and (6) woman who is pregnant, breastfeeding, or planning to become pregnant.

### Assessments

Demographics assessed at baseline included age, gender, education, employment, income, number of dependents, and marital status. The baseline survey also assessed smoking and quitting history for sample description. The 6-item Fagerström Test for Nicotine Dependence [40] was used to assess the degree of physical dependence on nicotine, and the 1-item Contemplation Ladder [39] was used to assess readiness to quit smoking.

Acceptability was assessed using measures of treatment utilization and satisfaction. The primary treatment utilization outcomes were the number of log-ins to the assigned website and time (days) from first to last use. Treatment satisfaction was assessed using 8 items on the 3-month outcome survey, with overall satisfaction being the primary satisfaction end point. The satisfaction survey also contained forced-choice response items assessing whether participants would recommend the program to a friend and their satisfaction with the program’s content, organization, and ease of use. Scores of at least “somewhat” on a 5-point Likert-type scale (ranging from “not at all” to “very much,” with “somewhat” in the middle) were coded as being satisfied for the purposes of cross-group comparisons. Open-ended questions inquired about what participants liked the most and the least about their assigned program. Acceptability measures were administered last to prevent unblinding of the intervention group assignment until the end of the study. We also explored acceptability of the Vet Flexiquit avatar (“Alex”) at the 3-month follow-up using 2 subscales of the Agent Persona Inventory [43], which contains items rated on a 1 (strongly disagree) to 5 (strongly agree) scale. The 2 subscales included were facilitating learning (10 items; eg, “Alex kept my attention”) and human-like (5 items; eg, “Alex showed emotion”). We also included a set of items assessing the avatar’s attributes, drawing in part from items on the Robotic Social Attributes Scale [44]. For these items, the avatar’s attributes were rated on a scale of 1 (definitely does not describe Alex) to 9 (definitely describes Alex) and included the following: pleasant, likable, agreeable, trustworthy, sincere, supportive, relatable, credible, scary, strange, awkward, and judgmental. We also included four open-ended study-specific questions to assess the acceptability of the avatar: (1) “Based on your experiences, what were the least important or least useful parts of Alex?” (2) “Based on your experiences, what were the most important or most useful parts of Alex?” (3) “What, if anything, would you change about Alex? Why?” and (4) “What additional feedback would you like to provide us about the avatar in the program?”

Efficacy for smoking cessation was assessed as the 7-day PPA at 3 months after randomization (“Have you smoked at all, even a puff, in the last 7 days?”), biochemically confirmed by saliva cotinine <30 ng/mL on an Alere iScreen test. Secondary efficacy end points included the following: (1) cotinine-confirmed 30-day point prevalence smoking abstinence at 3 months after the randomization, (2) self-reported and cotinine-confirmed 7-day and 30-day PPA from any nicotine or tobacco products other than Food and Drug Administration–approved cessation medications, and (3) increase in readiness to quit on the Contemplation Ladder. The use of all nicotine and tobacco products was measured at baseline and 3-month follow-up. For all end points involving biochemical verification, abstinence from smoking was limited to self-reporting for participants who were using other sources of nicotine (either therapeutic or nontherapeutic), as saliva cotinine testing cannot distinguish between smoked tobacco and other sources of nicotine. Saliva cotinine tests were sent by mail to veterans ahead of their 3-month follow-up visit conducted via telephone or VA-approved teleconferencing programs. Veterans were instructed on how to administer the test and provided photos of their test results to the study team as part of the call.

An assessment of preliminary efficacy for impacting ACT’s theory-based mechanism of change (ie, psychological flexibility) was measured at baseline and at the 3-month follow-up to evaluate changes in (1) acceptance of smoking triggers on the 27-item Avoidance and Inflexibility Scale (AIS) [43] and (2) valued living on the 10-item Valuing Questionnaire [45], which has 2 subscales representing values progress (5 items) and values obstruction (5 items). The use of other behavioral and pharmacological interventions for tobacco cessation was also assessed at the 3-month follow-up.

### Procedures

#### Overview

With the exception of some recruitment activities, the trial was conducted remotely, a modification to the original study procedures necessitated by the COVID-19 pandemic. At the beginning of the first telephone visit, participants were provided with detailed information about the study, and verbal consent was obtained following the guidelines of the VA Bedford Healthcare System Institutional Review Board. Following informed consent, participants completed the study procedures as outlined in the research plan. Veterans who were screened as ineligible to participate were provided with information about other resources to help them quit smoking.

Eligible participants were randomized using an automated algorithm via a custom web-based system at the baseline visit, which occurred approximately 1 week after screening and was conducted via telephone or a VA-approved conferencing program. A permuted block design with random blocks of sizes 2 and 4 was used to balance randomization on the stratification variable: high (>5) or low (≤5) readiness to quit on the Contemplation Ladder [39]. Participants received an email with a link to their assigned website and were guided through their first log-in by a research staff member. Investigators and participants were blinded to both allocation sequence and intervention assignment.

Participants were invited to complete a follow-up assessment of the primary and secondary trial outcomes at 3 months after randomization. All assessments were administered via telephone or VA-approved conferencing programs by trained study staff. At the 3-month follow-up, reminder calls were made 2 to 3 days before the scheduled appointment. At the end of the study, all participants received information on VA smoking cessation programs and resources (eg, SmokefreeVET text messaging program, QuitVET telephone quit line, Stay Quit Coach app, and in-person counseling).

#### Vet Flexiquit Content

Vet Flexiquit was designed as a web application that is optimized for viewing on a mobile screen, but it can also be accessed on other devices (eg, desktop or tablet). Consistent with the original Flexiquit program, Vet Flexiquit contains 6 sessions designed to be completed in order, spaced over a minimum of 3 days between sessions, with automated pacing and prompting from the program. Each session took approximately 10 to 25 minutes to complete. Vet Flexiquit also includes a 6-week SMS text messaging program to (1) prompt completion of the next session (ie, 6 messages—1 per session—alerting the user that a new session has unlocked, and up to 6 reminder messages—1 per session—if the session is not completed within 3 days of being unlocked) and (2) provide motivational and supportive intervention content (3-7 messages per week for 6 weeks). Because Vet Flexiquit is designed for smokers at all stages of readiness to quit smoking, prompts to reduce smoking and set a quit date do not occur until later sessions. At the end of the program, users were sent an email containing all session handouts.

Session 1 introduces the avatar coach, Alex, who provides an overview of the program and shares their own story of quitting. Users complete an interactive game to identify personal values that guide quitting and review quit stories from other veterans. Session 2 focuses on trigger awareness through web-based questions, graphs, pictures, and experiential exercises and metaphors, and it introduces the ACT concept of creative hopelessness, recognizing that efforts to control thoughts, feelings, or physical sensations related to smoking can be counterproductive. Session 3 addresses the topic of creative hopelessness and introduces cognitive defusion, that is, psychological distancing from thoughts—as an alternative to thought control. Session 4 completes the topic of cognitive defusion, encourages setting a quit date in the next week, and prompts users to practice defusing from thoughts that they will not be able to quit as part of quit planning. Session 5 starts with a reflection on the past week’s successes and difficulties, introduces the acceptance strategy of willingness as a means of handling smoking triggers, and covers relapse prevention via self-compassion and recommitment to quit. Session 6 also starts with a reflection on the past week’s successes and difficulties, reviews content from previous sessions, and ends with a video emphasizing the importance of letting go of the need to control internal experiences, such as thoughts, feelings, or physical sensations. Similar to SmokefreeVET, Vet Flexiquit includes information about smoking cessation medications and a link to the VA Crisis Line. As such, differential encouragement of nonstudy treatments was not a confounding factor in evaluating the novel content of Vet Flexiquit.

#### SmokefreeVET Content

The control intervention was the National Cancer Institute’s SmokefreeVET program. This web-based intervention was designed to promote smoking cessation among veterans by providing educational materials about cessation treatments; tools to cope with urges and relapse; ways to stay motivated; and brief tips for veterans with depression and anxiety, substance use disorders, HIV, and other physical and mental health problems. The content of the SmokefreeVET website is consistent with that of the United States Clinical Practice Guidelines [14] for tobacco treatment, which include heterogeneous techniques most closely aligned with cognitive behavioral therapy [46]. In addition to content differences, the structure of SmokefreeVET also differs from Vet Flexiquit in that all the content is accessible from the outset, and users choose which materials they wish to engage with. As the website is publicly available, a local copy was used for study purposes to prevent changes to the site while the study was ongoing and to mask the name of the site to prevent treatment contamination or bias. Links to the VA Quitline and other national resources were removed to prevent any confounding with other VA tobacco cessation resources. Similar to Vet Flexiquit, participants assigned to receive the SmokefreeVET website were also given a 6-week SMS text messaging program to motivate and support cessation. These messages were drawn from the SMS text message bank of the SmokefreeVET SMS text messaging program and delivered at the same frequency as the motivational and supportive messages in the Vet Flexiquit arm (ie, 3-7 messages per week for 6 weeks).

### Statistical Analysis Plan

As a pilot treatment development project, this study was not designed for power to detect statistically significant differences by treatment group. However, we explored outcomes of the 2 interventions and obtained a preliminary estimate of effect size. We planned a sample size of 50, which is consistent with the recommended sample size for pilot feasibility trials [46].

Participant demographics were described overall and by treatment arm using frequencies and percentages for categorical variables and means and SDs for continuous variables. Differences in baseline demographics by treatment arm were tested using the Fisher Exact Test and 1-way ANOVA for categorical and continuous variables, respectively. To compare Vet Flexiquit and SmokefreeVET on acceptability, preliminary efficacy, and impact on theory-based change processes at 3 months, we tested for statistical differences between arms using logistic regression (for binary outcomes) or negative binomial regression (for count outcomes) with adjustment for the stratification variable, baseline readiness to quit (high vs low), and adjustment for any baseline variables that had a statistically significant difference by study arm and were associated with the outcome (ie, potential confounders). Change score end points included changes in AIS scores and change in Contemplation Ladder scores. To compare change score end points (eg, change in treatment process measures) between study arms, we calculated the change score as the 3-month follow-up minus baseline score and used a linear regression model with adjustment for the baseline value of the measure of interest and for the stratification variable, baseline readiness to quit (high vs low), and adjustment for any baseline variables that had a statistically significant difference by study arm and were associated with the outcome. Satisfaction ratings with Vet Flexiquit’s avatar coach, Alex, were presented descriptively as frequencies and percentages. Statistical significance was based on a 2-sided α level of.05. SAS (version 9.4; SAS Institute) was used for all statistical analyses.

Consistent with the Russell standard for smoking cessation trials, participants with missing smoking data were considered nonabstinent [47] in the primary analysis of cessation outcomes. As a sensitivity analysis, we also reported the results of complete-case analyses. Treatment acceptability and process measures (ie, AIS scores) were analyzed using complete-case analysis, as there was no reasonable method of imputing these data.

### Ethics Approval

This study was conducted in accordance with the Declaration of Helsinki and approved by the VA Bedford Healthcare System Institutional Review Board (#1215233) and the Fred Hutchinson Cancer Center Institutional Review Board (IR# 10097). There was a complete discussion of the study with potential participants, and all participants provided written informed consent to participate in the study. To protect participants’ confidentiality, identifiable information collected as part of the study screening and enrollment procedures was stored in a VA shared drive that was not accessible to anyone outside the research team, and each participant was assigned a code that linked all of their data. Once participants were enrolled in the trial, a limited set of identifiers that were needed for the intervention to be implemented (ie, first name, email address, mobile telephone number, number of cigarettes smoked per day, and cost per pack of cigarettes) were entered into the secure study portal, which had a security feature that prohibited the subsequent retrieval of this information. The participants were compensated up to US $35 to complete all study assessments. Compensation was not linked to the completion of intervention activities. This trial was preregistered on ClinicalTrials.gov (NCT04502524).

## Results

### Screening and Enrollment

**Figure 1:**
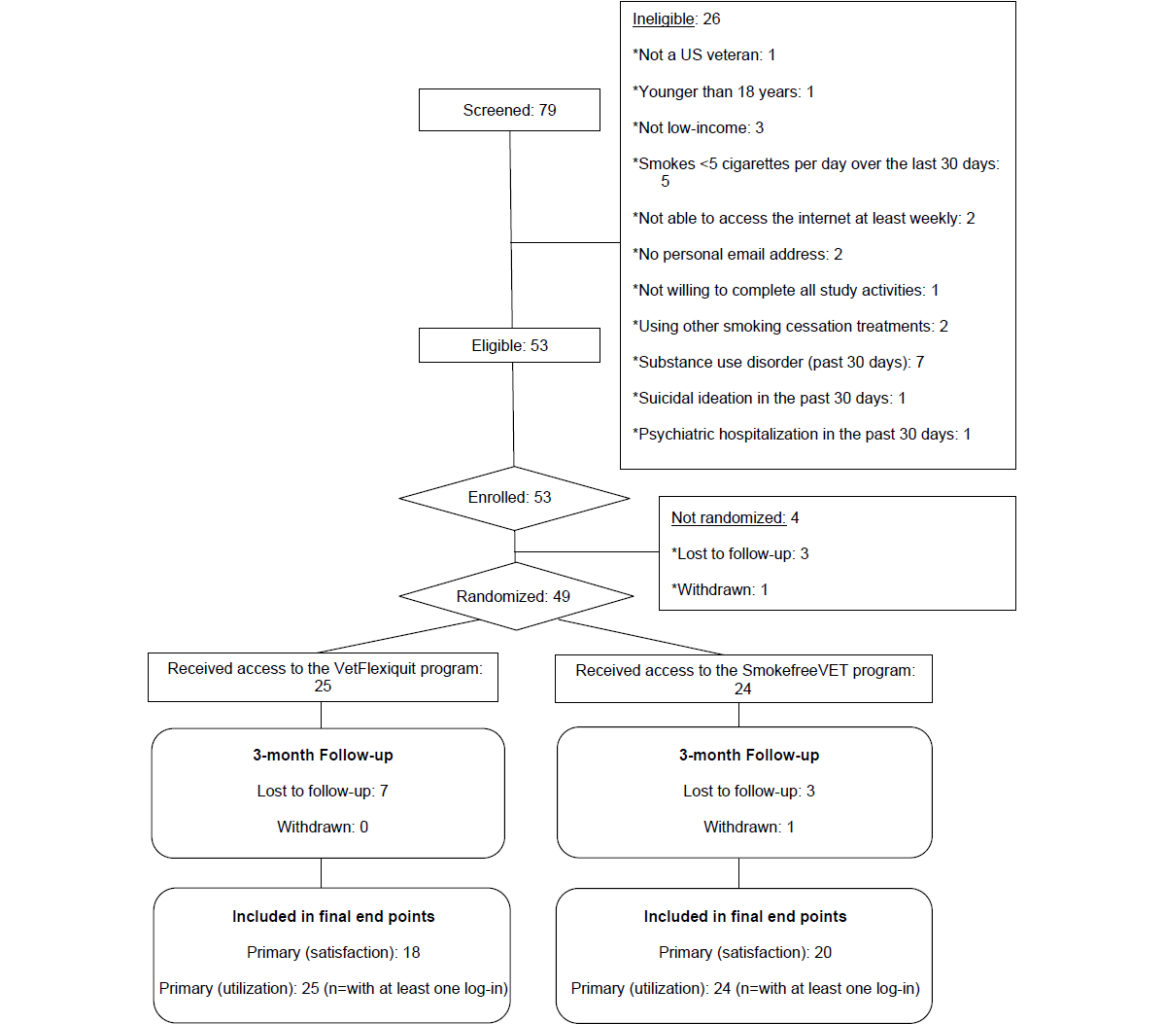
Participant flow diagram.

A total of 79 individuals engaged in a brief phone screen for this study, of whom 26 were screened as ineligible, with the most common reasons for ineligibility being smoking ≤5 cigarettes per day over the last 30 days (n=5) and meeting the criteria for an active substance use disorder within the past 30 days (n=7). In total, 53 participants were eligible and enrolled in the study. Four participants who were enrolled in the study were not randomized because they were lost to follow-up (n=3) or withdrew (n=1*)* before randomization. The final sample comprised 49 participants who were randomized between February 3 and October 22, 2021. See Figure 1 for the CONSORT (Consolidated Standards of Reporting Trials) diagram of the participant flow and see Multimedia Appendix 1 for the CONSORT-EHEALTH (Consolidated Standards of Reporting Trials of Electronic and Mobile Health Applications and Online Telehealth) checklist.

### Baseline Characteristics

Of the 49 veterans who were randomized, 25 received the Vet Flexiquit intervention and 24 received the SmokefreeVET intervention. Table 1 provides the baseline characteristics of the participants who were randomized, overall and by treatment group. Overall, the participants were 51.3 (SD 15) years of age (range 26-84), on average, and a majority identified as man/male (37/49, 76%), White (39/49, 80%), and non-Hispanic (41/49, 84%), with at least some college completed (39/49, 80%) and with an income ≤US $50,000 per year (38/49, 78%). Within the past year, 14% (7/49) reported being homeless and 49% (24/49) reported having at least a somewhat difficult time paying the bills. The majority of participants (39/49, 80%) self-reported having mental health conditions, including anxiety disorder (7/49, 14%), depression (12/49, 24%), bipolar disorder (4/49, 8%), posttraumatic stress disorder (13/49, 27%), schizophrenia (2/49, 4%), or another disorder (1/49, 2%). Participants smoked an average of 16.7 (SD 8.1) cigarettes at baseline and had an average Fagerström Test for Nicotine Dependence score of 4.3 (SD 2.2). Only a minority used e-cigarettes (6/49, 12%) or other forms of tobacco (6/49, 12%) in addition to smoking cigarettes.

**Table 1:** Baseline characteristics of the randomized sample.

		SmokefreeVET (n=24)	Vet Flexiquit (n=25)	Total (N=49)	*P* value^a^	
Age (years), mean (SD)		54.8 (16.1)	47.9 (13.3)	51.3 (15)	.11	
**Gender identity, n (%)**						.61
	Man/male	17 (71)	20 (80)	37 (76)		
	Woman/female	6 (25)	5 (20)	11 (22)		
	Transwoman/transfemale	1 (4)	0 (0)	1 (2)		
Hispanic/Latinx		5 (21)	3 (12)	8 (16)	.46	
**Race, n (%)**						.72
	American Indian/Alaska Native	0 (0)	2 (8)	2 (4)		
	Black or African American	2 (8)	2 (8)	4 (8)		
	White	20 (83)	19 (76)	39 (80)		
	Multiple	2 (8)	2 (8)	4 (8)		
**Education, n (%)**						>.99
	High school or less	5 (21)	5 (20)	10 (20)		
	At least some college	19 (79)	20 (80)	39 (80)		
**Income (US $), n (%)**						.74
	<$20,000 per year	6 (25)	5 (20)	11 (22)		
	$20,000-$49,999 per year	14 (58)	13 (52)	27 (55)		
	≥$50,000 per year	4 (17)	7 (28)	11 (22)		
Homeless in the past year, n (%)		2 (8)	5 (20)	7 (14)	.42	
Somewhat/very difficult time paying bills, n (%)		10 (42)	14 (56)	24 (49)	.40	
**Mental health conditions, n (%)**						.82
	Anxiety disorder	4 (17)	3 (12)	7 (14)		
	Depression disorder	5 (21)	7 (28)	12 (24)		
	Bipolar disorder	2 (8)	2 (8)	4 (8)		
	Posttraumatic stress disorder	7 (29)	6 (24)	13 (27)		
	Schizophrenia	0 (0)	2 (8)	2 (4)		
	Different than listed	0 (0)	1 (4)	1 (2)		
	None of the above	6 (25)	4 (16)	10 (20)		
FTND^b^ total score, mean (SD)		4.2 (2.1)	4.3 (2.3)	4.3 (2.2)	.86	
Cigarettes per day, mean (SD)		15.4 (7)	18 (9.1)	16.7 (8.1)	.27	
Any e-cigarette use, n (%)		3 (12)	3 (12)	6 (12)	>.99	
Any other tobacco use, n (%)		2 (8)	4 (16)	6 (12)	.67	
Quit confidence, mean (SD)		55.5 (31.2)	71.4 (18.1)	63.6 (26.3)	.03	
Quit readiness (Contemplation Ladder), mean (SD)		7.8 (2.1)	8 (2.3)	7.9 (2.2)	.79	
AIS^c^ thoughts, mean (SD)		3 (0.3)	3 (0.5)	3 (0.4)	.65	
AIS feelings, mean (SD)		3 (0.5)	3.1 (0.6)	3 (0.5)	.50	
AIS physical sensations, mean (SD)		3 (0.5)	3 (0.5)	3 (0.5)	.76	
VQ^d^ progress score, mean (SD)		22.2 (6.2)	22.3 (6.4)	22.3 (6.2)	.95	
VQ obstruction score, mean (SD)		11.5 (7.3)	15.0 (6.5)	13.3 (7.0)	.09	

^a^*P* values from Linear Model ANOVA (for continuous outcomes) or Fisher Exact Test (for count outcomes).

^b^FTND: Fagerström Test for Nicotine Dependence.

^c^AIS: Avoidance and Inflexibility Scale.

^d^VQ: Values Questionnaire.

Before conducting analyses comparing treatment groups on the primary and secondary trial end points, we evaluated whether there were any differences between the treatment arms at baseline that, despite occurring by chance, might bias outcome evaluations. The only characteristic that differed significantly by arm was quit confidence. Consequently, it was included as an additional covariate in cases where quit confidence was significantly correlated with the dependent variable in the model.

### Acceptability Outcomes

The 3-month follow-up was completed by 68% (17/25) of participants on the Vet Flexiquit arm and 79% (19/24) of participants on the SmokefreeVET arm. Satisfaction outcomes described subsequently only include those who completed the follow-up survey, whereas the utilization outcomes rely on objective tracking of website utilization and include all randomized participants.

The primary aim of the study was to compare Vet Flexiquit and SmokefreeVET in terms of acceptability outcomes of satisfaction and utilization at 3 months. The results are presented in Table 2. Regarding satisfaction, on the primary indicator of overall satisfaction, 100% (17/17) of Vet Flexiquit participants and 95% (18/19) of SmokefreeVET participants reported being satisfied with their assigned intervention. Differences between arms in overall satisfaction were not statistically significant nor were there differences in any of the other dimensions of satisfaction with the assigned treatment. On the primary utilization outcomes, the average number of log-ins for Vet Flexiquit was 3.7 (SD 3.6) and the average for SmokefreeVET was 3.2 (SD 4.1), with no significant difference between groups. There was also no significant difference by treatment arm in time from the first to last log-in, which averaged 18.7 (SD 27.3) days for Vet Flexiquit and 26.7 (SD 47.2) days for SmokefreeVET.

Examining qualitative data from the treatment satisfaction survey, themes that emerged from the comments of participants in both interventions were (1) the perceived value of support from peers or providers in addition to a digital intervention (eg, “Like your persistence, follow-up from staff” and “Nice to speak to someone”); (2) appreciation for the SMS text message component of the intervention (eg, “Text messages were key, They were good reminders of real life situations which were very relatable,” “Text messages raised my awareness,” and “Text messages served as a continuing reminder. Motivating.”), including a desire for more messages or for messages to continue for a longer duration (eg, “Wish there were more of them” and “Should have continued to have text messages for the duration of the program”); (3) the accessibility of the intervention (eg, “It is always available” and “Program availability was great”); and (4) being able to use the program at their own pace (eg, “Self-paced, no pressure” and “At my own pace and time”). Within the Vet Flexiquit arm, comments about the overall program included themes of (1) liking the ACT approach (eg, “Like that I have a way of learning to quit for good,” “Whole Health model compliant,” and “Liked exercises and psychology work on thoughts/emotions, awareness and priorities”) and (2) having a good experience with the avatar coach (eg, “I liked working with Alex,” “Like the avatar,” and “Felt like a friend”).

Qualitative data from Vet Flexiquit participants indicated that for those who only logged in once or twice, reasons for not using the program more include the following: time constraints (eg, “Time constraints prohibited me from using Vet Flexiquit as often as I would have liked”), the program was used in large increments at one time (eg, “I tended to binge my lessons”), and inability to get back on after losing their phone (“Not able to get back online after losing my phone”). For SmokefreeVET, the main reason for only using the program once or twice was the preference for in-person treatment (eg, “Biggest preference is face to face”). However, several participants in the SmokefreeVET arm indicated that they used the SMS text messages, even if they did not engage more in the web-based component of the program (eg, “Like the text messages, they came at the times when I really wanted to smoke”).

To better understand the experience of participants using the novel Vet Flexiquit program, we conducted several additional within-arm analyses to understand program use and satisfaction with its components (Table 3). In terms of session completion, the largest proportion of Vet Flexiquit participants (17/25, 68%) completed none of the 6 sessions, 4% (1/25) completed 3 sessions, 8% (2/25) completed 4 sessions, 4% (1/25) completed 5 sessions, and 16% (4/25) completed all 6 sessions. Among those who completed the follow-up survey, 50% (9/18) reported that they did not recall seeing Alex, the avatar, suggesting that they may not have meaningfully engaged with the web-based portion of Vet Flexiquit. Of those who recalled seeing Alex and provided ratings of the avatar, scores on the Agent Persona Inventory suggested positive impressions of Alex on the Facilitating Learning Subscale (mean 44.6, SD 76), with a possible score range of 10 to 50, and on the Human-like Subscale (mean 21, SD 3.3), with possible scores ranging from 5 to 25. On items rating Alex’s attributes on a 1 to 10 scale of agreement modeled after the Robotic Social Attributes Scale, including both positive (eg, trustworthy, motivating, and sincere) and negative (eg, scary, awkward, and judgmental) attributes, the average score for positive attributes suggested high agreement (mean 8.1, SD 1.1), with low agreement for negative attributes (mean 1.2, SD 0.3).

**Table 2:** Acceptability, cessation, and mechanism of change outcomes.

				SmokefreeVET (n=24)		Vet Flexiquit (n=25)		Estimate (95% CI)^a^		*P* value^a^
**Primary acceptability outcomes**										
	**Satisfied overall^b^**			n=19		n=17		N/A^c^		>.99
		Not at all or a little, n (%)	1 (5)		0 (0)					
		Somewhat or mostly or very much, n (%)	18 (95)		17 (100)					
	Number of log-ins^d^, mean (SD; range)			3.2 (4.1; 1 to 20)		3.7 (3.6; 1 to 12)		0.05 (−0.46 to 0.56)		.85
	Duration of use (days since first log-in)^d^, mean (SD; range)			26.7 (47.2; 0 to 185.6)		18.7 (27.3; 0 to 95)		−0.44 (−1.60 to 0.71)		.45
**Secondary acceptability outcomes**										
	**Program motivated to reduce or quit^e^**			n=20		n=18		0.39 (0.06 to 2.60)		.33
		Not at all or slightly, n (%)	2 (10)		4 (22)					
		Somewhat or moderately or extremely, n (%)	18 (90)		14 (78)					
	**Recommend to another veteran^e,f^**			n=20		n=18		0.46 (0.01 to 15.56)		.66
		Yes, n (%)	17 (85)		17 (94)					
		Unsure, n (%)	3 (15)		1 (6)					
	**Useful overall^e^**			n=19		n=17		0.83 (0.10 to 6.94)		.86
		Not at all or a little, n (%)	2 (11)		2 (12)					
		Somewhat or mostly or very much, n (%)	17 (89)		15 (88)					
	**Useful text messages^e,f^**			n=20		n=18		1.32 (0.16 to 10.65)		.79
		Not at all or a little, n (%)	5 (25)		2 (11)					
		Somewhat or mostly or very much, n (%)	15 (75)		16 (89)					
	**Made for someone like me^e,f^**			n=20		n=18		0.15 (0.01 to 4.95)		.29
		Not at all or a little, n (%)	3 (15)		2 (11)					
		Somewhat or mostly or very much, n (%)	17 (85)		16 (89)					
	**Easy to navigate^e^**			n=18		n=16		0.33 (0.02 to 4.59)		.41
		Not at all or a little, n (%)	1 (6)		2 (12)					
		Somewhat or mostly or very much, n (%)	17 (94)		14 (88)					
	**Gave me new ways of looking at quitting^e,f^**			n=20		n=17		2.70 (0.22 to 33.44)		.44
		Not at all or a little, n (%)	5 (25)		1 (6)					
		Somewhat or mostly or very much, n (%)	15 (75)		16 (94)					
**Cessation outcomes**										
	**7-day PPA^g^ from smoking at 3 months**									
		Cotinine-confirmed (SmokefreeVET: n=24; Vet Flexiquit: n=25; missing=smoking)^e^, n (%)	4 (17)		3 (12)		0.65 (0.13 to 3.32)		.60	
		Cotinine-confirmed (SmokefreeVET: n=20; Vet Flexiquit: n=18; complete case)^e^, n (%)	4 (20)		3 (17)		0.72 (0.13 to 3.91)		.70	
		Self-reported (SmokefreeVET: n=24; Vet Flexiquit: n=25; missing=smoking)^e,f^, n (%)	5 (21)		4 (16)		0.66 (0.15 to 2.90)		.58	
	**30-day PPA from smoking at 3 months**									
		Cotinine-confirmed (SmokefreeVET: n=24; Vet Flexiquit: n=25; missing=smoking)^e^, n (%)	3 (12)		3 (12)		0.94 (0.17 to 5.29)		.94	
		Self-reported (SmokefreeVET: n=24; Vet Flexiquit: n=25; missing=smoking)^e^, n (%)	3 (12)		3 (12)		0.94 (0.17 to 5.29)		.94	
	**7-day PPA from all nicotine or tobacco at 3 months**									
		Cotinine-confirmed (SmokefreeVET: n=24; Vet Flexiquit: n=24; missing=smoking)^e^, n (%)	3 (12)		2 (8)		0.61 (0.09 to 4.12)		.61	
		Self-reported (SmokefreeVET: n=24; Vet Flexiquit: n=25; missing=smoking)^e^, n (%)	4 (17)		3 (12)		0.46 (0.08 to 2.54)		.37	
	**Change in readiness to quit^g^**			n=19		n=18		0.56 (−1.13 to 2.26)		.50
		Values, mean (SD)	−0.5 (3.6)		−0.4 (3.3)					
	**Number of quit attempts^d^**			n=19		n=17		−0.73 (−1.46 to 0.003)		.51
		Values, mean (SD)	3.2 (6.7)		1.7 (1.1)					
**Mechanisms of change**										
	**AIS^i^ thoughts score change^h^**			n=20		n=18		0.10 (−0.40 to 0.60)		.69
		Values, mean (SD)	0.4 (0.7)		0.5 (0.7)					
	**AIS feelings score change^h^**			n=20		n=18		0.11 (−0.40 to 0.61)		.67
		Values, mean (SD)	0.3 (0.8)		0.4 (0.6)					
	**AIS physical sensations score change^h^**			n=20		n=18		−0.02 (−0.51 to 0.46)		.92
		Values, mean (SD)	0.5 (0.7)		0.5 (0.7)					
	**VQ^j^ progress change^h^**			n=19		n=18		1.31 (−2.17 to 4.78)		.45
		Values, mean (SD)	0.1 (7.1)		0.1 (6.5)					
	**VQ obstruction change^h^**			n=19		n=18		−0.05 (−3.95 to 3.85)		.98
		Values, mean (SD)	−2.7 (5.6)		−3.6 (6.3)					
**Concomitant treatment**										
	Used a smoking cessation medication (SmokefreeVET: n=20; Vet Flexiquit: n=18; excludes vaping or e-cigs)^e^, n (%)			16 (80)		10 (56)		0.32 (0.07 to 1.39)		.13
	Used nonstudy behavioral treatment (SmokefreeVET: n=20; Vet Flexiquit: n=18)^e^, n (%)			6 (30)		1 (6)		0.16 (0.02 to 1.59)		.12

^a^All the models included adjustments for baseline quit readiness (low or high), and effect sizes refer to the effect of Vet Flexiquit relative to SmokefreeVET as the reference group. Models with change score outcomes represent the 3-month follow-up score minus the baseline score and were also adjusted for the respective baseline scores.

^b^For overall satisfaction, a reliable estimate could not be calculated using a model, as all the responses were the same in the Vet Flexiquit arm; *P* value in the table is from a Fisher exact test.

^c^N/A: not applicable.

^d^Estimate and *P* value are from a negative binomial model.

^e^Estimate (odds ratio) and *P* values were obtained from a logistic regression model.

^f^Model also adjusted for baseline quit confidence.

^g^PPA: point prevalence abstinence.

^h^Estimate and *P* values were obtained from a linear regression model.

^i^AIS: Avoidance and Inflexibility Scale.

^j^VQ: Values Questionnaire.

**Table 3:** Secondary acceptability outcomes in the Vet Flexiquit arm assessed 3 months after randomization.

	Values, n	Values, mean (SD)	Values, median (range)
Agent Persona Inventory: Facilitate Learning Subscale	8	44.6 (7.6)	47.5 (27-50)
Agent Persona Inventory: Human-like Subscale	8	21 (3.3)	22 (16-25)
Alex Pleasant^a^	9	8.8 (0.4)	9 (8-9)
Alex Likable^a^	9	8.4 (0.7)	9 (7-9)
Alex Agreeable^a^	9	8.4 (0.7)	9 (7-9)
Alex Trustworthy^a^	8	7.4 (2.2)	8 (3-9)
Alex Sincere^a^	8	7.8 (1.8)	8.5 (5-9)
Alex Supportive^a^	8	8.8 (0.5)	9 (8-9)
Alex Relatable^a^	8	7.8 (2)	8 (3-9)
Alex Credible^a^	8	7.6 (2.1)	8.5 (3-9)
Alex Scary^a^	8	1.3 (0.7)	1 (1-3)
Alex Strange^a^	9	1.2 (0.4)	1 (1-2)
Alex Awkward^a^	9	1.4 (1)	1 (1-4)
Alex Judgmental^a^	9	1 (0)	1 (1-1)
Alex Motivating^a^	8	8 (1.4)	8.5 (5-9)
Alex Entertaining^a^	9	7.3 (2.7)	9 (1-9)
Positive descriptors of Alex—average^a^	9	8.1 (1.1)	8.2 (5.6-8.9)
Negative descriptors of Alex—average^a^	9	1.2 (0.3)	1 (1-2)

^a^Alex (avatar) descriptors were rated on a Likert-type scale ranging from 1 (not at all) to 9 (extremely).

### Cessation Outcomes

The cessation outcomes are shown in Table 2. On the outcome of cotinine-confirmed 7-day PPA at 3 months, the quit rates for Vet Flexiquit (3/25, 12%) and SmokefreeVET (4/24, 17%) were not significantly different. Similarly, there were no statistically significant differences when using other definitions of cessation (eg, self-report, abstinence from all nicotine and tobacco, and 30-day PPA). Regarding the outcome of change in Contemplation Ladder scores between baseline and the 3-month follow-up, there was also no significant difference between the groups.

### ACT’s Theory-Based Change Processes

The outcomes of the change process are listed in Table 2. On the AIS, changes in acceptance of thoughts (mean 0.5, SD 0.7 for Vet Flexiquit; mean 0.4, SD 0.7 for SmokeFreeVET), feelings (mean 0.4, SD 0.6 for Vet Flexiquit; mean 0.3, SD 0.8 for SmokefreeVET), and physical sensations (mean 0.5, SD 0.7 for Vet Flexiquit; mean 0.5, SD 0.7 for SmokefreeVET) did not differ significantly by arm. Similarly, on both the Values Progress and Values Obstruction subscales of the Values Questionnaire, changes were in a similar direction and not significantly different across arms.

### Concomitant Treatment

In post hoc analyses, we examined differences in the use of concomitant tobacco cessation treatment by study arm to evaluate the potential impact on cessation outcomes. The use of nonstudy pharmacotherapy (10/18, 56% in the Vet Flexiquit arm vs 16/20, 80% in the SmokefreeVET arm; odds ratio 0.32, 95% CI 0.07-1.39) and behavioral treatment (1/18, 6% in the Vet Flexiquit arm vs 6/20, 30% in the SmokefreeVET arm; odds ratio 0.16, 95% CI 0.02-1.59) were both descriptively lower among Vet Flexiquit participants than among SmokefreeVET participants, although the differences were not statistically significant.

### Relationship Between Smoking Abstinence and Program Use

In another post hoc exploratory analysis, we examined the relationship between the number of log-ins to the assigned program and smoking abstinence. Because of the small number of participants in each arm who achieved biochemically confirmed abstinence, we examined these outcomes descriptively. The 3 Vet Flexiquit quitters logged in 7, 8, and 10 times, and all of them completed all 6 sessions. In addition, none of the Vet Flexiquit quitters participated in any other quit program. The 4 SmokefreeVET quitters logged in 2, 3, 4, and 5 times. One of the SmokeFree quitters participated in another quit program at the same time.

## Discussion

### Principal Findings

The aim of this study was to evaluate the acceptability of the novel Vet Flexiquit program relative to SmokefreeVET for socioeconomically disadvantaged veterans, with the secondary aim of exploring the effects on cessation and theory-based change processes. Overall, treatment satisfaction was very high for both programs, with 100% (17/17) of Vet Flexiquit and 95% (18/19) of SmokefreeVET participants indicating that they were satisfied with the interventions at 3-month follow-up. Utilization was similar across the arms. Qualitative data indicated that participants liked the SMS text messaging component of both interventions and that veterans would have liked more messages because they found them to be motivating and good reminders of intervention principles and exercises. The participants also indicated that they liked how accessible their assigned program was. Given that (1) only 4% of veterans enrolled in VHA care who use tobacco engage in tobacco cessation counseling programs within a given year [13] and (2) socioeconomically disadvantaged veterans may experience both travel costs and scheduling difficulties in participating in tobacco cessation programs at VHA facilities, digital interventions that can be used at any time may result in an increase in participation in tobacco cessation interventions for socioeconomically disadvantaged veterans.

In the Vet Flexiquit arm, only 16% (4/25) of the participants completed all 6 sessions, which is lower than that in previous studies using the same foundational program for sexual and gender minority young adults in the United States (where 39% completed all 6 sessions [41]) and for young adults in Cyprus (where 57% completed all 6 sessions [32]). In addition, 68% (17/25) of the participants in the Vet Flexiquit arm did not complete the first session based on objective utilization data. Although it was not possible to determine from the utilization data how much of the first session these participants completed before intervention attrition, 50% (9/18) of those who completed the 3-month outcome survey did not recall seeing Alex, the avatar, which is a central feature of the program introduced at the beginning of the first session. As all randomized participants completed their first program log-in with the support of a study staff member, these data suggest that half of the participants never meaningfully engaged in the program outside of accessing it as part of a study visit. It is likely that this failure to engage meaningfully with the program also occurred in the SmokefreeVET arm, but owing to differences in how the programs are structured and how the users’ actions were tracked, it was not possible to determine how many participants in that arm failed to engage beyond the initial log-in. Less-frequent use of a digital intervention in this study relative to prior work [32,41] may, in part, be owing to the differences in ages between veterans in this sample (with a mean age of 51, SD 15 years) and previous samples of young adults aged 18 to 30 years. Issues of technology literacy may affect interest in and ability to navigate the program among older users, as older veterans tend to report that digital interventions are more difficult to use [48].

Despite limited engagement in the Vet Flexiquit program, users indicated that they liked the overall ACT approach and having an avatar coach as a guide in the program. Qualitative data also indicated that veterans wanted interactions with professionals or peers as part of both programs, as evidenced by comments about being grateful for the interactions with the research staff. Although this makes scalability more challenging, this preference is in alignment with the VHA-wide strategy of integrating digital health tools into clinical care. The VHA has already rolled out innovative programs to facilitate the adoption of these tools in practice, including the VHA mobile health ambassadors program, which trains clinical staff to provide education about and support to veterans for digital interventions and improving the integration of digital interventions in VHA care. Integrated care, combining provider instruction and check-in with an intervention such as Vet Flexiquit, could also improve engagement and outcomes for digital tobacco cessation interventions, along the lines of positive outcomes for integrated tobacco cessation counseling in VHA mental health care [47]. Future research should evaluate strategies for integrating digital interventions for tobacco cessation into VHA clinical care models.

Regarding the secondary aims that explored tobacco cessation outcomes and impact on theory-based mechanisms of change, on all these end points, there was no evidence of significantly different outcomes for the 2 interventions. Inadequate exposure to the content of the program is a potential reason for the failure of Vet Flexiquit to show greater impact on these outcomes. In the post hoc analysis, all the participants who quit smoking in the Vet Flexiquit arm logged into the program between 7 and 10 times, and all of them completed all 6 sessions of the program, suggesting that higher engagement may improve outcomes. Another potential reason for the lack of difference between treatment arms is that the proportion of participants in the control arm receiving concomitant treatment was descriptively higher than that in the Vet Flexiquit arm for both pharmacotherapy (80% vs 56%) and other behavioral support (30% vs 6%), which may have boosted cessation outcomes in the control arm. Finally, veterans who participated in this study likely differ in numerous ways from young adults in prior studies testing other versions of Flexiquit [32,41], including not only their age but also their socioeconomically disadvantaged status and a very high rate of mental health comorbidity (ie, 39/49, 80%). The quit rate for Vet Flexiquit in this study was very similar to that of a different web-based ACT program evaluated for individuals with bipolar disorder, most of whom were recruited from VHA settings but with the requirement that they be ready to quit within 30 days of study enrollment [31]. Using that previous study as a benchmark, a similar quit rate for a program that enrolls veterans in the VHA at all levels of quit readiness is a promising finding. The 12% rate of 30-day PPA at 3 months in both the Vet Flexiquit and SmokefreeVET arms exceed the 30-day PPA rate of 4.5% at 3 months for the SmokefreeVET SMS text messaging program in a previous study [16], which is also promising.

### Limitations

The results of this study should be interpreted considering several limitations. First, as is typical for pilot studies where the goal is to establish feasibility rather than to detect statistically significant differences between arms, the sample size was small and follow-ups were limited to short-term outcomes. A finding of no difference in the comparison between 2 active treatments in a small pilot trial should not be interpreted to mean that no difference exists. Larger studies on Vet Flexiquit with longer-term outcomes are necessary to better understand the efficacy of the intervention, either alone or in combination with peer or professional support. Some ACT interventions have demonstrated greater effects at long-term follow-up, possibly because additional time and practice may be needed for consolidation to occur when presented with new, counterintuitive information [26,49]. This study was also conducted during the COVID-19 pandemic, which represents a unique context in which to study the effects of tobacco treatment. Changes in smoking attributable to pandemic-related shifts in social and occupational activities as well as changes in physical and mental health [50] may have influenced both the willingness and ability to engage with a digital cessation program as well as the likelihood of smoking cessation. The study design was also affected by the pandemic, transforming a primarily in-person design into a fully remote one. However, this study also had several strengths, including (1) biochemical verification of abstinence, albeit with the limitation that cotinine tests could not be completed for participants who reported quitting smoking but still using other forms of nicotine (n=2; in total, 1 participant in each arm reported abstinence that could not be verified biochemically owing to other nicotine use); (2) a good retention rate compared with other tobacco treatment trials conducted within the VHA [51,52], particularly during the COVID-19 pandemic, which was associated with an increase in tobacco use among veterans during the pandemic period [50]; (3) blinding of the control condition to reduce expectancy effects on study findings; and (4) the use of an active treatment comparison arm instead of waitlist control, as in previous work [32].

### Conclusions

Although preliminary, these results suggest that Vet Flexiquit and SmokefreeVET have similar acceptability and potentially efficacy as tobacco cessation digital interventions for socioeconomically disadvantaged veterans. Participants found both programs to be acceptable and accessible, and the inclusion of SMS text messages in both programs and an avatar coach in the Vet Flexiquit were found to be helpful. Overall, it appears that veterans prefer having professional support integrated with digital intervention use. On the basis of this feedback, a professionally supported digital program integrated with pharmacotherapy could be more acceptable and effective in VHA health care settings than stand-alone digital treatments, as long as veterans are willing to engage with a multicomponent program. For those who are not, the availability of a stand-alone digital cessation program offers an alternative, accessible option for assistance with quitting. Further research is needed to optimize engagement with Vet Flexiquit, to develop and evaluate a professionally supported version of Vet Flexiquit, and to evaluate the utility of Vet Flexiquit in clinical practice, either as a stand-alone program or when integrated into clinical care.

## Appendix

Multimedia Appendix 1 CONSORT-EHEALTH checklist (V 1.6.1).

## Abbreviations

ACT: Acceptance and Commitment Therapy
AIS: Avoidance and Inflexibility Scale
CONSORT: Consolidated Standards of Reporting Trials
CONSORT-EHEALTH: Consolidated Standards of Reporting Trials of Electronic and Mobile Health Applications and Online Telehealth
EQQUAL: Empowered, Queer, Quitting, and Living
PPA: point prevalence abstinence
SES: socioeconomic status
VHA: Veterans Health Administration

## Data Availability

The deidentified, anonymized data set generated for this study is available from MMK upon reasonable request. Those requesting data will be asked to sign a Data Use Agreement and comply with the Department of Veterans Affairs and non–Department of Veterans Affairs privacy regulations.
